# Cancer incidence and prevalence in cystic fibrosis patients with and without a lung transplant in France

**DOI:** 10.3389/fpubh.2022.1043691

**Published:** 2022-11-22

**Authors:** Christine Rousset-Jablonski, Faustine Dalon, Quitterie Reynaud, Lydie Lemonnier, Clémence Dehillotte, Flore Jacoud, Marjorie Berard, Marie Viprey, Eric Van Ganse, Isabelle Durieu, Manon Belhassen

**Affiliations:** ^1^Université Claude Bernard Lyon 1, Research on Healthcare Performance (RESHAPE), INSERM U1290, Lyon, France; ^2^Department of Internal Medicine, Cystic Fibrosis Center, Hospices Civils de Lyon, Groupe Hospitalier Sud, Lyon, France; ^3^Département de chirurgie, Centre Léon Bérard, Lyon, France; ^4^PELyon, PharmacoEpidemiologie Lyon, Lyon, France; ^5^Vaincre la Mucoviscidose Association, Paris, France; ^6^Health Data Department, Hospices Civils de Lyon, Lyon, France; ^7^Respiratory Medicine, Croix-Rousse University Hospital, Lyon, France

**Keywords:** cystic fibrosis, transplantation, cancer incidence, claims data, national registry

## Abstract

**Background:**

Cystic fibrosis (CF) care and the life expectancy of affected patients have substantially improved in recent decades, leading to an increased number of patients being diagnosed with comorbidities, including cancers. Our objective was to characterize the epidemiology of cancers between 2006 and 2017 in CF patients with and without a lung transplant.

**Methods:**

Medical records of CF patients from 2006 to 2016 in the French CF Registry were linked to their corresponding claims data (SNDS). The annual prevalence and incidence rates of cancers were estimated from 2006 to 2017 in CF patients without lung transplant and in those with lung transplant after transplantation.

**Results:**

Of the 7,671 patients included in the French CF Registry, 6,187 patients (80.7%) were linked to the SNDS; among them, 1,006 (16.3%) received a lung transplant. The prevalence of any cancer increased between 2006 and 2017, from 0.3 to 1.0% and from 1.3 to 6.3% in non-transplanted and transplanted patients, respectively. When compared to the general population, the incidence of cancer was significantly higher in both non-transplanted [Standardized Incidence Ratio (SIR) = 2.57, 95%CI 2.05 to 3.17] and transplanted (SIR = 19.76, 95%CI 16.45 to 23.55) patients. The median time between transplant and the first cancer was 3.9 years. Among the 211 incident cancer cases, the most frequent malignant neoplasms were skin neoplasm (48 cases), lung cancers (31 cases), gastro-intestinal (24 cases), and hematologic cancers (17 cases).

**Conclusion:**

The overall burden of cancer in CF patients is high, particularly following lung transplantation. Therefore, specific follow-up, screening and cancer prevention for CF patients with transplants are necessary.

## Introduction

During recent decades, the life expectancy of people with cystic fibrosis (pwCF) has increased dramatically, and treatment with cystic fibrosis transmembrane regulator (CFTR) modulators will likely contribute to further improvements. In countries with well-developed cystic fibrosis (CF) care, more than half of pwCF followed in CF centers are adults (1, 2). However, along with this increased life expectancy, new complications have emerged, such as extra-respiratory comorbidities and cancer. Indeed, several studies have reported a greater than expected number of digestive tract cancers, particularly in the small intestine, colon, and biliary tract (3, 4). The pathogenesis of cancer in CF remains unclear, but inflammation and the role of CFTR have been discussed (5, 6). Specifically, regarding the risk of cancer for pwCF, two different situations have to be identified: pwCF who have undergone lung transplantation (LT) and those who have not.

Despite such major improvements in CF care, LT remains necessary for patients with severe CF as the ultimate option. In Italy, Spain, France, the UK and the US, between 3 and 11% of CF patients receive a LT (2, 7, 8). Despite better survival for pwCF than for patients receiving LT for other indications (due to their young age at transplantation), several US epidemiological studies have shown an increased risk of cancer for pwCF with transplants (3, 4, 9, 10), but European data are lacking.

In the literature, a particular risk of digestive tract cancers is described for non-transplanted pwCF (11). In 2013, in the US, non-transplanted pwCF were also found to be at higher risk of testicular cancer and chronic lymphocytic leukemia than the general US population (3). To the best of our knowledge, one observational study has been published in Europe, showing an increased risk for kidney, thyroid, endocrine cancers, lymphoma and non-melanoma skin cancers in Swedish patients, and another recent study described a higher risk of cancer, mainly gastrointestinal (GI), skin, breast and hematological cancers in the UK CF population (12, 13).

With recent epidemiological changes in pwCF, data need to be actualized to further discuss the necessity of specific follow-up and screening and even more for lung-transplanted pwCF. This paper characterizes the epidemiology of cancer in pwCF and incidence trends from 2006 to 2017 in France according to patient status with respect to LT.

## Materials and methods

### Data sources

The French CF Registry collects clinical data for all pwCF followed at CF care centers, including transplanted patients (2).

In the SNDS, the following anonymized data are prospectively recorded for all French patients covered by national health insurance: sociodemographic characteristics, date of death, out-of-hospital reimbursed healthcare expenditures (from both public and private healthcare), and hospital discharge summaries with International Classification of Diseases (ICD-10) codes, and direct information on medical diagnoses for patients who have full coverage by the NHS for all medical expenses [Chronic Disease status], including patients diagnosed with CF in France (14).

In this study, data for each patient of the CF registry were individually linked to the French administrative health care database (SNDS), using a probabilistic method (15).

### Study population and study design

This study involved patients recorded in the French CF Registry between 2006 and 2016 and for whom linkage with the SNDS could be achieved. Patients were followed until December 31, 2017.

Two subgroups were defined: (1) Patients with a PLT were considered those with a medical procedure or hospital diagnosis for LT between 2006 and 2017 in the SNDS and those with a LT recorded in the CF registry before 2006. (2) Patients without any medical procedure or hospital diagnosis related to LT, either in the SNDS or in the CF registry, even before 2006. Patients with any history of other transplantation (mainly kidney, liver) were excluded from both groups.

Patients with a first diagnosis of invasive cancer (identified with ICD-10 codes from hospital admissions, Supplementary Table 1) were identified in the two groups of pwCF according to their LT status. Their incidence status was defined by the absence of chronic disease status for cancer in the SNDS before 2006 or by the absence of cancer recorded in the CF registry before 2006.

The index date was the 01/01/2006 for patients born prior to 2006, and the birth date recorded in the registry for patients born after 2006.

For non-transplanted patients, follow-up started at the index date and finished at the end of follow-up, as defined by the following events, whichever occurred first: death, loss to follow-up (date of last information recorded in the SNDS prior to a 24-month period without any reimbursement), or end of the study period in the SNDS (31/12/2017). For PLT patients, follow-up started at the PLT date, or from the index date if PLT occurred before 2006, to the end of follow-up, as defined for the other group.

### Data analysis

Socio-demographic characteristics and clinical data are presented using descriptive statistics, as follows: for quantitative variables, the sample size (N), mean, standard deviation (std), median, and first and third quartiles (Q1–Q3) are reported; for qualitative and ordinal variables, the sample size (N) and frequency are reported.

Comorbidities were identified using chronic disease status, hospital diagnosis, or specific therapies.

Cases of cancer were classified as prevalent or incident. Prevalent cancer cases were defined by at least one hospitalization and/or a chronic disease status for cancer for a given cancer in a given year. Incident cancer cases were defined by a first hospitalization and/or a first chronic disease status for cancer for a given cancer in a given year. The incidence rate (per 1,000 person-years) was defined as the total number of new cancer cases (the numerator) divided by the sum of the person-time of the at-risk population (the denominator). It was estimated for each studied year, and only patients included in the studied year were counted in the denominator, that is, patients with at least 1 day of follow-up in that year. So long as a patient did not have any cancer yet, he was still considered as at risk of developing it and therefore contributed to the denominator.

We used the standardized incidence ratio (SIR), defined as the ratio of the number of cancers observed to the number of cancers expected, and we calculated 95% confidence intervals (CIs) for the SIR while assuming that the observed cases of cancer followed a Poisson distribution. The number of cancers expected during the at-risk period was determined by applying age- (within 5-year groups), sex-, and calendar year-specific incidence rates obtained from the data published by the French National Cancer Registry in 2019 (based on data from 2018) to the total person-years accumulated in the corresponding categories [16].

The time to onset of the first cancer was plotted using a cumulative incidence curve, allowing mortality to be taken into account as a competitive risk when the mortality rate was >10%.

The statistical analysis was performed with SAS Enterprise Guide^®^ (SAS Institute, North Carolina), version 7.13.

### Ethics

This observational study was conducted using anonymized data after approval by the French Institute for Health Data (approval n° 217, on December 1, 2016) and the National Informatics and Liberty Committee (approval n° DE-2018-001, on March 12, 2018).

## Results

This study considered 7,671 patients included in the French CF Registry between 2006 and 2016. Probabilistic linkage with the claims database could be performed for 6,187 (80.7%) patients. Among them, 1,006 (16.3%) patients had a PLT, and 5,074 were non-transplanted patients (107 remaining patients have been transplanted from other organs, mainly kidney, liver). Table 1 presents the sociodemographic and clinical characteristics of PLT and non-transplanted pwCF.

**Table 1:** Sociodemographic and clinical characteristics of non-transplanted and PLT pwCF.

**Sociodemographic and clinical characteristics**	**Non-transplanted patients** **(*N* = 5,074)**	**PLT patients (post-transplant period of follow-up)** **(*N* = 1,006)**
Female	2,405 (47.4%)	518 (51.5%)
**Time of follow-up (in year)**		
Mean (std)	10.8 (2.5)	5.4 (4.0)
Median (Q1–Q3)	12.0 (11.6–12.0)	4.8 (1.9–8.6)
**Age at the end of follow-up (in year)**		
0–8	662 (13.0%)	0 (0%)
9–14	955 (18.8%)	10 (1.0%)
15–19	774 (15.3%)	62 (6.2%)
20–24	692 (13.6%)	100 (9.9%)
25–29	637 (12.6%)	191 (19.0%)
30–34	468 (9.2%)	215 (21.4%)
35–39	333 (6.6%)	158 (15.7%)
40–45	236 (4.7%)	142 (14.1%)
>45	317 (6.2%)	128 (12.7%)
**Age at the end of follow-up (in year)**		
Mean (std)	22.6 (13.5)	33.5 (9.9)
Median (Q1–Q3)	20.0 (12.0–30.0)	33.0 (26.0–40.0)
Min–Max	0.0–87.0	10.0–65.0
**Age at the date of transplantation (in year)**		
0–8	–	5 (0.5%)
9–14	–	43 (4.3%)
15–19	–	122 (12.1%)
20–24	–	243 (24.2%)
25–29	–	227 (22.6%)
30–34	–	172 (17.1%)
35–39	–	97 (9.6%)
40–45	–	62 (6.2%)
>45	–	35 (3.5%)
**Age at the date of transplantation (in year)**		
Mean (std)	–	27.5 (8.9)
Median (Q1–Q3)	–	26.0 (21.0–33.0)
Min–Max	–	5.0–60.0
**Death**	176 (3.5%)	266 (26.4%)
**Comorbidities identified by hospitalization diagnosis or specific therapies**		
Diabetes	1,188 (23.4%)	922 (91.7%)

### Cancers in non-transplanted pwCF

During follow-up, 1.8% of non-transplanted pwCF had 1 to 3 cancers, with a mean age at first cancer of 35.2 (±19.5). However, the annual prevalence of cancers in this population increased with time, from 0.3% in 2006 to 1.0% in 2017 (Table 2).

**Table 2:** Annual prevalence of cancer in non-transplanted and PLT patients.

	**2006**	**2007**	**2008**	**2009**	**2010**	**2011**	**2012**	**2013**	**2014**	**2015**	**2016**	**2017**
**Non-transplanted patients**												
Number	4,126	4,242	4,384	4,516	4,610	4,703	4,761	4,783	4,827	4,863	4,876	4,865
Annual number of cancer cases	13	17	22	15	20	18	25	25	29	33	38	46
Prevalence (%)	0.32	0.40	0.50	0.33	0.43	0.38	0.53	0.52	0.60	0.68	0.78	0.95
**Transplanted patients after PLT**												
Number	210	257	302	351	404	465	519	591	637	666	727	763
Annual number of cancer cases after PLT	9	12	19	16	20	19	22	30	24	38	46	48
Prevalence (%)	4.3	4.7	6.3	4.6	5.0	4.1	4.2	5.1	3.8	5.7	6.3	6.3

**Figure 1:**
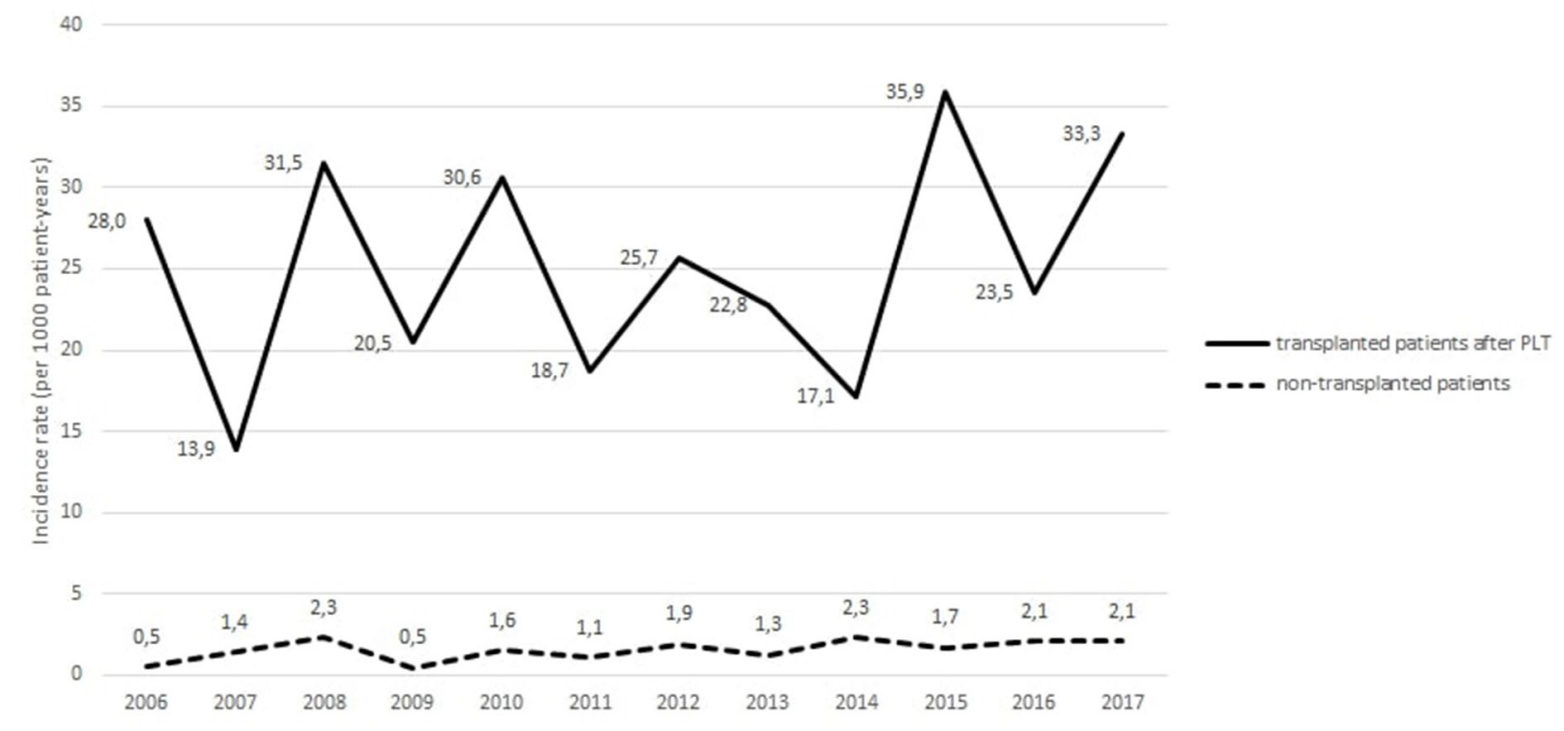
Temporal trends in the annual incidence rates of cancers (all sites included) occurring in non-transplanted patients and in transplanted patients after lung transplantation.

Among non-transplanted patients, 86 had a first cancer during their follow-up. Figure 1 depicts the annual incidence rates of cancer between 2006 and 2017 in non-transplanted patients. Incidence rates were stable over time, from 0.5 to 2.3 per 1,000 patient-years. Table 3 presents the number of observed and expected cancers with corresponding SIR and 95%CI values among non-transplanted patients for the period from 2006 to 2017. For the 54,425 person-years of observation during the period from 2006 to 2017, 86 invasive cancers were reported in non-transplanted CF patients compared to 33.5 expected cancers in the general population (SIR = 2.57, 95%CI 2.05 to 3.17) (Table 3). There was a significantly higher number of colorectal cancer (SIR = 4.41, 95%CI 1.62 to 9.59) and of lung cancer (SIR = 4.96, 95%CI 2.57 to 8.67) in non-transplanted CF patients than in the general population.

**Table 3:** Observed and expected numbers of cancers and SIRs among non-transplanted and PLT patients with CF in France from 2006 to 2017.

	**Non-transplanted patients**			**Transplanted patients after PLT**		
**Site (ICD-10 code)**	**Observed N incident cancers**	**Expected N cancers in general population**	**SIR (95% CI)**	**Observed N incident cancers**	**Expected N cancers in general population**	**SIR (95%CI)**
All cancers	86	33.52	2.57 (2.05 to 3.17)	125	6.32	19.76 (16.45 to 23.55)
Oesophagus	2	0.27	7.47 (0.90 to 26.97)	0	0.05	0.00 (0.00 to 64.22)
Stomach	0	0.37	0.00 (0.00 to 8.04)	3	0.08	**37.76 (7.79 to 110.35)**
Small intestine	1	0.06	16.95 (0.43 to 94.41)	4	0.01	**317.53 (86.52 to 812.99)**
Colorectal	6	1.36	**4.41 (1.62 to 9.59)**	6	0.23	**26.45 (9.71 to 57.58)**
Pancreas	1	0.55	1.80 (0.05 to 10.05)	3	0.10	**30.79 (6.35 to 90.00)**
Lip, oral cavity and pharynx	4	1.21	3.30 (0.90 to 8.44)	4	0.24	**16.97 (4.62 to 43.46)**
Nasal cavities, paranasal sinuses, middle and inner ear	0	0.06	0.00 (0.00 to 47.17)	1	0.01	**81.07 (2.05 to 451.71)**
Larynx	0	0.20	0.00 (0.00 to 14.84)	1	0.04	26.06 (0.66 to 145.22)
Lung	12	2.42	**4.96 (2.57 to 8.67)**	19	0.47	**40.36 (24.30 to 63.03)**
Bone sarcoma	2	0.30	6.62 (0.80 to 23.92)	1	0.02	**42.63 (1.08 to 237.54)**
Malignant melanoma and skin cancers^*^	9	2.13		39	0.54	
Soft tissue sarcoma	1	0.57	1.76 (0.04 to 9.81)	1	0.08	11.77 (0.30 to 65.58)
Breast	8	5.51	1.45 (0.63 to 2.86)	1	1.6	0.62 (0.02 to 3.48)
Cervix uteri	2	0.55	3.61 (0.44 to 13.03)	3	0.19	**16.19 (3.34 to 47.31)**
Corpus uteri	1	0.42	2.37 (0.06 to 13.19)	0	0.06	0.00 (0.00 to 53.19)
Ovary	1	0.50	2.01 (0.05 to 11.18)	0	0.09	0.00 (0.00 to 33.11)
Prostate	2	1.37	1.46 (0.18 to 5.27)	1	0.12	8.29 (0.21 to 46.16)
Testis	1	1.79	0.56 (0.01 to 3.12)	3	0.39	**7.62 (1.57 to 22.28)**
Kidney	2	1.15	1.74 (0.21 to 6.30)	4	0.22	**18.29 (4.98 to 46.84)**
Bladder	1	0.41	2.46 (0.06 to 13.73)	3	0.06	**51.40 (10.60 to 150.20)**
Central nervous system (CNS)	0	1.40	0.00 (0.00 to 2.14)	2	0.19	**10.77 (1.30 to 38.92)**
Thyroid gland	2	2.63	0.76 (0.09 to 2.75)	1	0.69	1.44 (0.04 to 8.03)
Hodgkin's lymphoma	3	1.50	1.99 (0.41 to 5.83)	3	0.22	**13.58 (2.80 to 39.69)**
Non-Hodgkin lymphoma	2	3.12	0.64 (0.08 to 2.31)	5	0.37	**13.68 (4.44 to 31.92)**
Acute myeloid leukemia	2	0.57	3.54 (0.43 to 12.77)	1	0.07	14.72 (0.37 to 82.06)
Chronic myeloid leukemia (LMC)	1	0.12	8.65 (0.22 to 48.22)	0	0.02	0.00 (0.00 to 125.31)

No cancer were observed for vulva, vagina, penis, uveal melanoma, chronic myelomonocytic leukaemia, liver and, intrahepatic bileduct, gallblader and extrahepatic bileduct.

*Expected number of cancers includes only melanoma. We thus could not calculate SIR for this entity.

### Cancers occurring after LT

Among PLT patients, 14.8% developed at least one cancer during the follow-up period (either before or after the transplantation). After transplantation, 13.7% had a diagnosis of one to five cancers, with a mean age at first cancer of 33.8 (±10.5). The annual prevalence of CF patients with any cancer occurring after PLT increased from 4.3% in 2006 to 6.3% in 2017 (Table 2).

Among PLT patients, 18 had cancer before PLT; 982 had no cancer before PLT. Among these 982 patients, 125 had a first cancer occurring after PLT. The incidence trends of cancer after PLT among transplanted patients are illustrated in Figure 1. No clear trend emerged over time, as incidence range were 28.0 per 1,000 patient-years in 2006, 18.7 per 1,000 patient-years in 2011, and 33.3 per 1,000 patient-years in 2017.

During 4,861 person-years of observation during the period from 2006 to 2017, 125 invasive cancers were reported in PLT patients after transplantation compared with 6.32 expected cancers (SIR = 19.76, 95%CI 16.45 to 23.55) (Table 3). There was a statistically higher number of stomach (SIR = 37.76, 95%CI 7.79 to 110.35), small intestine (SIR = 317.53, 95%CI 86.52 to 812.99) colorectal (SIR = 26.45, 95%CI 9.71 to 57.58) and pancreas cancers (SIR = 30.79, 95%CI 6.35 to 90.00), cancers of the lip, oral cavity and pharynx (SIR = 16.97, 95%CI 4.62 to 43.46), cervical (SIR = 16.19, 95%CI 3.34 to 47.31), testicular (SIR = 7.62, 95%CI 1.57 to 22.28), kidney (SIR = 18.29, 95%CI 4.98 to 46.84) and bladder cancers (SIR = 51.40, 95% CI 10.60 to 150.20), lung cancers (SIR = 40.36, 95%CI 24.30 to 63.03), cancers of the CNS (SIR = 40.36, 95%CI 24.30 to 63.03), Hodgkin (SIR = 13.58, 95%CI 2.80 to 39.69), and non-Hodgkin lymphomas (SIR = 13.68, 95%CI 4.44 to 31.92) after transplantation for PLT patients than in the general population.

**Figure 2:**
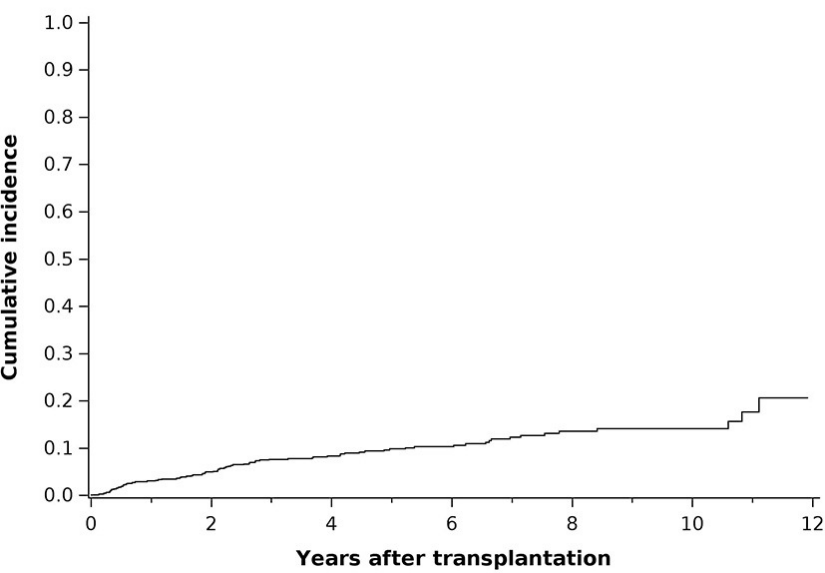
Cumulative incidence curve of cancers (all sites included) occurring after transplantation in PLT patients.

The median time from transplantation to the first cancer was 3.9 years. The probability of having any cancer at 10 years after transplantation was estimated to be 13.7% (Figure 2).

## Discussion

The linkage between French CF Registry data and the SNDS claims data confirmed that the overall burden of cancer in CF patients is high, particularly following LT. Comparison of SIR in French pwCF with those in the general population (16) confirmed higher incidence rates in French CF patients, as previously described both in the US and the UK CF population (3, 4, 9, 10, 12).

In our study, gastro-intestinal (GI) tract cancer was one of the most documented among CF patients, as previously shown in non-transplanted and in transplanted patients (3, 4, 9, 12, 17). We were not able to calculate separately SIR for colon or rectal cancers as both are counted as colorectal cancer in the French National Cancer Registry. This observation has led to specific GI cancer screening guidelines for CF patients: systematic colonoscopy with CF-specific intensive bowel preparation should be initiated at age 40 years (18), and CRC screening for organ transplant recipients with CF should be initiated earlier, at the age of 30 years, within 2 years of transplantation (18).

### Cancer risk in transplanted patients

Our study showed higher incidence rates of cancer in LT pwCF than in non-transplanted CF patients, thus confirming previous data (3, 4, 9, 10, 12, 17). Our study investigated a larger spectrum of cancer that previous studies. Indeed, in other studies, mainly GI tract cancer risk and lymphoma risk have been described among transplanted CF patients (9). In addition to the higher incidence of GI cancers in transplanted patients, we found a statistically significant higher incidence of cancers of lip, oral cavity and pharynx, cervical cancer, testicular, kidney and bladder cancers, lung cancer, CNS cancers, and Hodgkin and non-Hodgkin lymphoma.

There are several explanations for the higher incidence of cancer among transplanted patients. Transplantation itself may be responsible for this difference, being associated with an increased risk for cancers (18). Other factors, such as older age and the more severe clinical status of the transplanted group (high number of comorbidities), might play a role (19). Immunosuppression may also increase the risk of developing cancer in transplant patients through multiple mechanisms: the risk of oncoviral-driven malignancy due to long-term immunodeficiency, impaired immunosurveillance of neoplastic cells due to the non-specific mode of action of the majority of immunosuppressive drugs, and the pro-oncogenic properties of some immunosuppressive treatments (20, 21). The higher incidence of some cancers in PLT patients may also be due to reinforced cancer screening after PLT, such as cervical cancer screening, which is recommended annually for PLT patients and only every 3 to 5 years for non-transplanted patients (22, 23). Screening for skin cancer and for GI cancer is also recommended (18, 24).

Delay after transplantation should also be highlighted. The median time from transplantation to the first cancer was 3.9 years, a shorter delay than that described recently in the UK CF population. Archangelidi et al. (12) described an average time to first report of cancer of 7.1 years after transplantation. The risk of developing cancer has been shown to increase with the number of post-transplant years due to different factors: age at transplantation and aging during follow-up and the duration of exposure to immunosuppressive therapy. A comparison of our population with other transplanted populations to identify specific cancer risk related to CF might have been interesting but would have been difficult to achieve, as lung transplantation occurs in young patients in CF compared to other transplanted populations.

### Cancer risk in non-transplanted patients

We found an overall higher cancer incidence among non-transplanted pwCF compared to the non-transplanted general population, as described in the literature (3, 4, 9). Previous studies have also suggested a higher incidence of specific cancers among non-transplanted patients compared to the general population, such as lymphoma and thyroid cancers, testicular cancer and lymphoid leukemia (4). Our findings did not confirm these data, and we only found a higher incidence of GI tract cancers, and of lung cancers.

This last finding is contradictory to previous studies reporting a low incidence of cancer of the respiratory tract in CF patients (25), possibly attributable to the low prevalence of smoking reported in this population (12).

We observed a high frequency of melanoma and skin cancers among non-transplanted patients, whereas studies published in the 1990s have suggested a possible protective role for CFTR in CF patients without transplant on melanoma (26, 27). Different findings have been discussed to explain this protection regarding melanoma: a possible genetic protection of the F508 del mutation during melanoma genesis and a reduced time to risk exposure for melanoma in children with CF due to the reduced time spent outdoors (3). However, our data could not be compared to the general population as non-melanoma skin cancers are excluded from cancer registries, and number of expected cancers calculated concerns only melanoma.

### Pathogenesis of cancer in patients with CF

The pathogenesis of cancer in pwCF remains unclear, but multiple hypotheses have been proposed, especially for GI cancers. The specific role of *CFTR* in the physiopathology of GI tract cancer for pwCF has been evoked. The mechanisms underlying the actions of *CFTR* as a tumor suppressor are not clearly understood. Dysregulation of Wnt/β-catenin signaling and disruption of intestinal stem cell homeostasis and intestinal barrier integrity, as well as intestinal dysbiosis, immune cell infiltration, stress responses, and intestinal inflammation, have all been reported in human CF patients and in animal models (11). Lowered expression of *CFTR* is reported in sporadic CRC, and downregulation of *CFTR* is associated with poor survival (11). Furthermore, the enhanced risk for CRC extends to millions of people (more than 10 million in the US) who are heterozygous carriers of *CFTR* gene mutations (11). Chronic local inflammation and chronic increased GI epithelial cell turnover beginning in infancy and early childhood in CF, may also play a role (28, 29). Additionally, an inadequate immune response and intestinal stem cell regulation have been described in CFTR-knockout mice, which may offer some explanation (30).

Not specific to gastrointestinal cancers, hypotheses are proposed to explain the risk of cancer in general. Frequent exposure to radiation (i.e., X-rays and CT scans) might contribute to the increased risk of cancer in pwCF (31). The association between radiation exposure during childhood and cancer has been described for childhood acute lymphoblastic leukemia and solid tumors (32).

CF patients face some comorbidities that can influence cancer risk. CF-related diabetes was recently identified as an additional cancer risk factor, and a specific GI cancer screening among patients with CF-related diabetes has even been suggested (12). Unfortunately we were not able to make a specific analysis of the risk of cancer in the presence or absence of comorbidities. A link between diabetes or some other comorbidity and cancer risk could indeed explain some of our results as the over-representation of some cancers.

### Strenghts and limitations

Among the strengths of our study, the population-based study design, and the linkage with the national CF registry should be highlighted. This design allowed us to have an exhaustive collection of cancer cases in this context of rare disease. Some limitations of our study must be acknowledged, the most important of which is the underrepresentation of children younger than 7 years. Indeed, as the linkage used the dates of spirometry and as spirometry is seldom performed before the age of 6, many younger patients could not be linked (15). For similar reasons, the linkage led to an underrepresentation of deceased patients, as it was based on the death status, which is adequately identified only for patients affiliated with general health insurance (15). Underrepresentation of children might lead to overestimation of the cancer incidence and prevalence. However, very few cases of cancer are expected among children. Underrepresentation of deceased patients may have led to an underestimation of the number of cancer cases; it may also have been offset by overestimation of lung transplanted patients, as date of transplant was used for the linkage. Cancer cases were those declared in the SNDS, but we did not have histological confirmation. This could have led to a non-differential classification bias. The number of cancers expected was calculated using calendar year-specific incidence rates obtained from the data published by the French National Cancer Registry in 2019 (based on data from 2018) (16). This may have underestimated the difference from the general population as incidence of cancers has mainly increased over time. However, for some types of cancer (larynx and stomach in particular), this could have increased the difference observed in relation to the general population, as their incidence has decreased over time in the general population. Cases of melanoma and of skin cancers could not be differentiated in CF patients, as the identification code in SNDS is common. It prevented us from comparison with the general population for all skin cancers, as non-melanoma skin cancers are excluded from cancer registries. The absence of comparison of our population with other transplanted populations without CF to identify specific cancer risk related to CF can also be cited as a limitation.

In the future, the use of CFTR modulators may influence cancer risk among CF patients.

## Conclusion

This study highlights the high prevalence and incidence of cancer in CF patients. It also shows the necessity of specific follow-up and prevention for CF patients who have undergone transplantation and the need for resource allocation and training in future multidisciplinary adult CF teams with a comprehensive understanding of oncology.

## Data availability statement

The raw data supporting the conclusions of this article will be made available by the authors, without undue reservation.

## Ethics statement

This observational study was conducted using anonymized data after approval by the French Institute for Health Data (approval n° 217, on December 1, 2016) and the National Informatics and Liberty Committee (approval n° DE-2018-001, on March 12, 2018). Written informed consent from the participants legal guardian/next of kin was not required to participate in this study in accordance with the national legislation and the institutional requirements.

## Author contributions

CR-J, ID, QR, MBel, and FD contributed substantially to the study design and interpretation of the results, and were responsible for producing the initial draft of the paper. MBer and FJ carried out the statistical analysis and including figures and tables. EV, MV, LL, and CD contributed to the data interpretation and provided written comments, and feedback during manuscript development. All authors contributed to the article and approved the submitted version.

## Funding

This study was supported by the Vaincre La Mucoviscidose association that participated in providing data from their registry in the interpretation of data and in the writing of the report, and by the Comité de la Loire de la Ligue Contre le Cancer (Appel d'offres régional Auvergne-Rhône-Alpes et Saône-et-Loire de la Ligue Contre le Cancer 2018).

## Conflict of interest

Authors FJ, FD, MBer, and MBel are full-time employees of PELyon. EV is the scientific advisor and shareholder of PELyon. The remaining authors declare that the research was conducted in the absence of any commercial or financial relationships that could be construed as a potential conflict of interest.

## Publisher's note

All claims expressed in this article are solely those of the authors and do not necessarily represent those of their affiliated organizations, or those of the publisher, the editors and the reviewers. Any product that may be evaluated in this article, or claim that may be made by its manufacturer, is not guaranteed or endorsed by the publisher.

## Abbreviations

CF: cystic fibrosis
CFTR: cystic fibrosis transmembrane regulator
CI: confidence interval
CRC: colorectal cancer
ICD: international classification of diseases
LT: lung transplant
PLT: primary lung transplant
pwCF: people with cystic fibrosis
SIR: standardized incidence ratio
SNDS: système national des données de santé
(French national health data system).
