# Supplementary material for: Cancer incidence and prevalence in cystic fibrosis patients with and without a lung transplant in France
Source: Front Public Health. 2022 Nov 22;10:1043691. doi: 10.3389/fpubh.2022.1043691 (PMC9723348; doi:10.3389/fpubh.2022.1043691)
Supplement: Supplementary file 1 [file Table_1.DOCX]

**Supplementary material**

**Table 1.** ICD-10 codes used to identify the cancer cases.

| Neoplams | ICS-10 codes |
| --- | --- |
| Malignant neoplasms of lip, oral cavity and pharynx | C00-C14 |
| Malignant neoplasm of oesophagus | C15 |
| Malignant neoplasm of stomach | C16 |
| Malignant neoplasm of small intestine | C17 |
| Malignant neoplasm of colon | C18 |
| Malignant neoplasm of pancreas | C25 |
| Malignant neoplasms of respiratory and intrathoracic organs | C30-C39 |
| Malignant neoplasms of bone and articular cartilage | C40-C41 |
| Melanoma and other malignant neoplasms of skin | C43-C44 |
| Malignant neoplasms of mesothelial and soft tissue | C45-C49 |
| Malignant neoplasm of breast | C50 |
| Malignant neoplasms of female genital organs  Malignant neoplasms of male genital organs | C51-C58, C60-C63 |
| Malignant neoplasms of urinary tract | C64-C68 |
| Malignant neoplasms of eye, brain and other parts of central nervous system | C69-C72 |
| Malignant neoplasms of thyroid and other endocrine glands | C73-C75 |
| Malignant neoplasms, stated or presumed to be primary, of lymphoid, haematopoietic and related tissue | C81-C96 |
